# PSMA PET for the Evaluation of Liver Metastases in Castration-Resistant Prostate Cancer Patients: A Multicenter Retrospective Study

**DOI:** 10.3390/cancers14225680

**Published:** 2022-11-18

**Authors:** Susanna Mattoni, Andrea Farolfi, Fabio Formaggio, Gabriel Bruno, Paola Caroli, Juliano Julio Cerci, Matthias Eiber, Wolfgang Peter Fendler, Rita Golfieri, Ken Herrmann, Federica Matteucci, Cristina Mosconi, Giulia Paolani, Miriam Santoro, Lidia Strigari, Cristina Nanni, Paolo Castellucci, Stefano Fanti

**Affiliations:** 1 Nuclear Medicine, Alma Mater Studiorum University of Bologna, 40126 Bologna, Italy; 2 Division of Nuclear Medicine, IRCCS Azienda Ospedaliero-Universitaria di Bologna, 40138 Bologna, Italy; 3 Fundación Centro Diagnóstico Nuclear (FCDN), Buenos Aires C1417, Argentina; 4 IRCCS Istituto Romagnolo per lo Studio dei Tumori (IRST) “Dino Amadori”, 47014 Meldola, Italy; 5 Department of Nuclear Medicine, Quanta Diagnóstico e Terapia, Curitiba 80045-170, Brazil; 6 Department of Nuclear Medicine, Klinikum Rechts der Isar, Technical University Munich (TUM), 81675 Munich, Germany; 7 Department of Nuclear Medicine, University of Duisburg-Essen and German Cancer Consortium (DKTK)-University Hospital Essen, 45147 Essen, Germany; 8 Department of Radiology, IRCCS Azienda Ospedaliero-Universitaria di Bologna, 40138 Bologna, Italy; 9 Department of Medical Physics, IRCCS Azienda Ospedaliero-Universitaria di Bologna, 40138 Bologna, Italy

**Keywords:** CRPC, positron emission tomography (PET), prostate cancer, PSMA, radiomics

## Abstract

**Simple Summary:**

Visceral involvement in prostate cancer (PCa) represents a negative prognostic factor. Liver metastases typically occur in systemic, late-stage, castration-resistant prostate cancer (CRPC). The diagnostic performance of [68Ga]Ga-PSMA-11-PET for visceral metastases of CRPC patients has never been systematically assessed. Our aim was to evaluate the diagnostic performance of PSMA-PET compared to conventional imaging, i.e., CT or MRI, or liver biopsy in the detection of liver metastases in CRPC patients. The secondary aim was to assess the ability of radiomics to predict the presence of liver metastases. Regarding liver metastases assessment in CRPC patients, [68Ga]-PSMA-11-PET demonstrated moderate sensitivity while high specificity, positive predictive value, and reproducibility compared to conventional imaging and liver biopsy. However, nuclear medicine physicians should carefully assess the liver parenchyma on PET images, especially in patients at higher risk for liver metastases and with high PSA values. Moreover, radiomic features may aid in recognizing higher-risk patients to develop them.

**Abstract:**

Background: To evaluate the diagnostic performance of PSMA-PET compared to conventional imaging/liver biopsy in the detection of liver metastases in CRPC patients. Moreover, we evaluated a PSMA-PET/CT-based radiomic model able to identify liver metastases. Methods: Multicenter retrospective study enrolling patients with the following inclusion criteria: (a) proven CRPC patients, (b) PSMA-PET and conventional imaging/liver biopsy performed in a 6 months timeframe, (c) no therapy changes between PSMA-PET and conventional imaging/liver biopsy. PSMA-PET sensitivity, specificity, positive predictive value (PPV), negative predictive value (NPV), and accuracy for liver metastases were calculated. After the extraction of radiomic features, a prediction model for liver metastases identification was developed. Results: Sixty CRPC patients were enrolled. Within 6 months before or after PSMA-PET, conventional imaging and liver biopsy identified 24/60 (40%) patients with liver metastases. PSMA-PET sensitivity, specificity, PPV, NPV, and accuracy for liver metastases were 0.58, 0.92, 0.82, 0.77, and 0.78, respectively. Either number of liver metastases and the maximum lesion diameter were significantly associated with the presence of a positive PSMA-PET (*p* < 0.05). On multivariate regression analysis, the radiomic feature-based model combining sphericity, and the moment of inverse difference (Idm), had an AUC of 0.807 (95% CI:0.686-0.920). Conclusion: For liver metastases assessment, [68Ga]Ga-PSMA-11-PET demonstrated moderate sensitivity while high specificity, PPV, and inter-reader agreement compared to conventional imaging/liver biopsy in CRPC patients.

## 1. Introduction

Prostate cancer (PCa) is considered the second most frequently diagnosed cancer in men, and the prognosis is mainly determined by the presence or absence of metastases [1]. In 2016, the Prostate Cancer Clinical Trials Working Group 3 (PCWG3) summarized CRPC clinical trial recommendations, defining five clinical CRPC target populations based on the pattern of spread, ranging from non-metastatic to lymph nodes, bone disease or visceral metastatic CRPC, because this is prognostic [2]. Liver metastases typically occur in systemic, late-stage, castration-resistant prostate cancer (CRPC). According to autopsy studies, the most frequent sites of metastases are bone, lung, and liver and regarding the latter, the post-mortem detection reported of hepatic metastases is much higher as compared to in vivo detection [3,4,5]. Liver biopsy is the gold standard for confirmation of potential hepatic metastases, even if it is invasive and associated with tumor seeding and other complications. Multiphase contrast-enhanced Computed Tomography (ceCT) and Magnetic Resonance Imaging (MRI) are thus routinely employed to assess the liver when the suspicion of hepatic metastases occurs. Positron emission tomography (PET) tracers for PCa imaging, such as choline and fluciclovine, show physiological liver uptake, preventing an accurate evaluation of the liver itself. PET with small-molecule ligands that bind to cell-surface prostate-specific membrane antigen (PSMA-PET) has been introduced and received FDA approval [6]. PSMA-PET further localized metastases in more than half of patients with non-metastatic CRPC by conventional imaging, i.e., bone scintigraphy, ceCT, or MRI, and showed concordance in more than two third of PCWG3 clinical subtypes when compared to conventional imaging [7,8]. In normal organs, moderate uptake of [68Ga]Ga-PSMA-11 was found in the liver [9], while respiratory motion during PET acquisition may lead to blurring in resulting liver images [10]. All this entails consequent underestimation of tracer uptake within the hepatic parenchyma. Moreover, there is evidence that in PCa, liver metastases are frequently associated with dedifferentiation and high-grade cancers, leading to loss of PSMA expression with consequent obstacles to liver assessment [4]. Damjanovic et al. retrospectively evaluated the imaging characteristics of liver metastases in [68Ga]Ga-PSMA-11 PET [11]. After screening 739 PCa patients, 18 were found with hepatic involvement for a total of 80 PSMA-positive (77.7%) and 23 PSMA-negative (22.3%) metastases, suggesting that PSMA-PET precisely detects liver metastases in the majority of patients. The identification of new and reliable semi-quantitative and quantitative imaging parameters (e.g., using radiomic analysis) might be crucial to better identify PCa patients with liver metastases and worse prognoses. Radiomics is a new innovative, and a rapidly evolving bioinformatic approach to medical image analysis. Through the use of standardized mathematical-based models, radiomic features capture tissue and lesion characteristics and may, alone or in combination with other data, be used for clinical problem solving [12,13]. The aim of the study was to assess the diagnostic performance of PSMA-PET in the detection of liver metastases in a cohort of CRPC patients. Moreover, we sought to evaluate the inter-reader agreement when assessing the liver and a PSMA-PET/CT-based radiomic model to predict the presence of liver metastases.

## 2. Materials and Methods

### 2.1. Study Design and Participants

**Figure 1:**
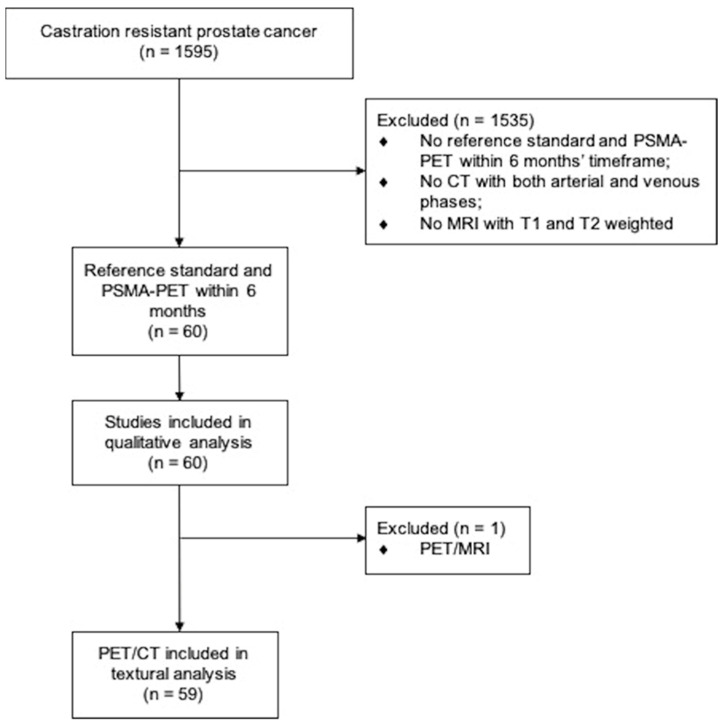
Consort diagram flow for patient selection.

Datasets from 1595 patients at 6 participating PET Centers (Bologna and Meldola [Italy], Essen and Munich [Germany], Buenos Aires [Argentina], and Curitiba [Brazil]) were retrospectively screened, including thirty patients from one retrospective study [8]. Inclusion criteria were: (a) documented CRPC during continuous ADT or previous lines of therapy for CRPC (physician note); (b) PSMA-PET within 6 months of reference standard defined as ceCT or MRI or liver biopsy; (c) no changes of therapy between the PSMA-PET and reference standard; (d) no other known type of cancer except for PCa. The reference standard was defined as the presence of one of the following: multi-phase ceCT or MRI including the liver in the field of view or a PET-guided liver biopsy. The patient’s flow is demonstrated in Figure 1. Approval was obtained by the IRCCS Azienda Ospedaliero-Universitaria di Bologna (244/2016/O/Oss). All patients gave written consent to undergo PSMA-PET and liver biopsy. The prerequisite to obtaining informed consent for inclusion in this retrospective analysis was waived by the ethics committee. All procedures performed in studies involving human participants were in accordance with the ethical standards of the institutional and/or national research committee and with the 1964 Helsinki declaration and its later amendments or comparable ethical standards.

### 2.2. PSMA-PET Imaging Procedures

PSMA-PET imaging. [68Ga]Ga-PSMA-11 PET was acquired in accordance with the international guideline as part of a PET/CT (*n* = 59) or PET/MRI (*n* = 1) examination [14]. In the case of PET/CT examination, a full-dose CT scan was acquired from the vertex to the mid-thigh. Automatic dose modulation was applied with a tube voltage of 120 kV (200–240 mAs). PET/MRI was performed on an integrated 3 Tesla PET/MRI system using high-channel surface coils. The field of view was from the skull base to the mid-thigh, and the protocol consisted of first a simultaneous PET and 3D Dixon VIBE sequences for scatter correction, a diffusion-weighted sequence with b-values of 50, 500, and 1000, then a standardized wbMRI protocol including an axial T1-weighted VIBE sequence after administration of gadolinium.

### 2.3. Reference Standard Procedures

#### 2.3.1. CT Imaging

Three-phase contrast-enhanced full-dose computed tomography (ceCT) was performed. CeCT included an unenhanced, arterial, and venous phase and late or equilibrium phase acquisitions carried out during the same examination. For all the protocols, 100–150 mL (based on the patient’s weight) of low-osmolar contrast agent was administered intravenously.

#### 2.3.2. MRI Imaging

Magnetic resonance imaging (MRI) included a hepatic arterial, a portal venous, and equilibrium phase acquisitions after intravenous injection of Gd-EOB-DTPA. Both dynamic and hepatobiliary phase images were obtained using a fat-suppressed 3D gradient-echo (GRE) sequence before and after intravenous bolus administration of Gd-EOB-DTPA.

#### 2.3.3. Liver Biopsy

Patients were positioned according to the lesion location and biopsy planning. A coaxial guide needle was inserted under the guidance of CT fluoroscopic imaging. One bed position PET/CT images were acquired to confirm the correct position of the coaxial needle (directing the needle towards the most accessible areas of high PSMA uptake) [15]. Four to six specimens were collected under CT fluoroscopy, fixed in 10% formalin, and sent for histopathological examination.

### 2.4. Image Interpretation

Anonymized PSMA-PET datasets were evaluated independently by three experienced nuclear medicine physicians with at least five years of experience in PSMA-ligand PET (PC, AF, and CN) who followed recent recommendations and the PROMISE molecular imaging TNM system [14,16]. Any area of increased focal uptake of [68Ga]Ga-PSMA-11 in PET distinctively above normal heterogeneity of liver background, any area of absent uptake, and any area of heterogeneous uptake, e.g., areas with perimetral high uptake and central decrease uptake, were screened. CT and MRI liver datasets were read separately and independently after anonymization by two radiologists with at least five years of experience in liver imaging (RG, CM). All readers were blinded to medical history. OsiriX MD (Pixmeo SARL, Bernex, Switzerland) was used for the central readings. Consensus (positive vs. negative) was determined by a majority vote for PSMA-PET, CT, and MRI. In case of disagreement for CT and MRI, a consensus was reached between the two readers.

### 2.5. Radiomic Extraction

Delineation of regions of interest (ROIs) for radiomic analysis was performed for PSMA-PET/CT by a nuclear medicine physician employing MIM Software (MIM Software Inc.; Beachwood, OH, USA), followed by confirmation of a second physician. Disagreement was resolved by consensus. The liver was delineated independently in every slice using the ROI tool both for PSMA-PET and for the CT part of the PET/CT examination. The pyradiomics module (Python v.3.8) was used for the extraction of 105 radiomic features per modality from both CT and PSMA-PET images using the liver ROIs [17].

### 2.6. Statistical Analysis

For continuous data, mean ± SD, median, and inter-quartile range (IQR) were reported, while categorical variables were described using absolute and relative (%) frequencies. Inter-observer agreement was determined by Fleiss’ κ and interpreted by the criteria of Landis and Koch [18]. Due to an asymmetric distribution of PSA, SUVmax, number of liver metastases, and maximum diameter of liver metastases, their association with PSMA-PET positivity was assessed with the non-parametric Mann-Whitney Test. Univariate analysis on both clinical and radiomic features was performed to evaluate the correlation with the outcome. In order to select only non-correlated radiomic features, a correlation analysis was performed for features extracted from CT and PSMA-PET, assuming a cut-off > 0.6. Thus, LASSO regression was employed to identify the independent predictive features of patients’ outcomes. A generalized linear model (GLM) was trained to predict the outcome, and ROC analysis was used to assess the prediction capability. Confidence intervals were calculated with the bootstrap method with 1000 replications. Statistical analysis was performed using R 4.0.2, and a *p*-value lower than 0.05 was considered significant.

## 3. Results

### 3.1. Patient Characteristics

Sixty CRPC patients were included among six recruiting PET Centers. Patient characteristics are given in Table 1. The median serum PSA level at the time of PSMA-PET was 6.3 ng/mL (IQR 1.6–45.3 ng/mL). The reference standard was made up of ceCT, MRI, and liver biopsy in 32/60 (53%), 19/60 (32%), and 9/60 (15%) patients, respectively. The median time between PSMA-PET and reference standard was 1 month (IQR 0–5). For patients validated through liver biopsy, 5/9 (56%) received the biopsy procedure within 10 days from the PSMA-PET date. Regarding previous therapies, 37/60 (62%) patients were abiraterone/enzalutamide/apalutamide naïve and 47/60 (78%) chemotherapy naïve; 12/60 (20%) were previously treated with PSMA radioligand therapy with [177Lu]Lu-PSMA.

### 3.2. Lesion Detection

#### 3.2.1. Reference Standard

**Figure 2:**
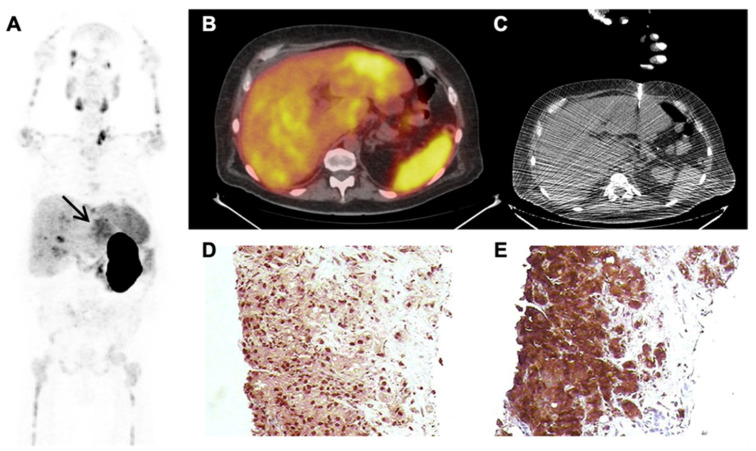
Case example of a PSMA-PET-guided liver biopsy. A 53-year-old man diagnosed with high-risk PCa treated with radical prostatectomy and pelvic lymph node dissection in 2015. After four years of biochemical relapse and progression under hormonal therapy with a diagnosis of CRPC. He underwent a PSMA-PET in March 2019 with a PSA of 75.0 ng/mL. PSMA uptake was demonstrated in pelvic and retroperitoneal lymph nodes, multiple bones, and within the liver (**A**,**B**). With known PET information, the patient underwent a liver biopsy two days later (**C**). Histopathology confirmed metastatic adenocarcinoma of the prostate positive for NK3.1 ((**D**), 400×) and PSA ((**E**), 400×).

Overall, ceCT, MRI, and liver biopsy identified 24/60 (40%) patients with liver metastases, and their distribution by liver segments is illustrated in Figure S1. In detail, liver metastases were detected in 11/32 (34%) with ceCT, in 7/19 (37%) with MRI, and in 8/9 (89%) with liver biopsy. Histopathology at biopsy was metastatic adenocarcinoma from PCa in 6/9 (67%) (case example in Figure 2), neuroendocrine metastases in 2/9 (22%), and focal hepatitis without malignant cells in 1/9 (11%). Liver metastases and ISUP Grade Group, PSA at the time of PET, and the number of previous lines of therapy for CRPC are displayed in Figure S2.

#### 3.2.2. PSMA-PET

Based on independent reads, PSMA-PET localized PCa in 55/60 patients, resulting in a 92% overall detection rate, and 48/60 (80%) were M1 patients, i.e., with distant metastases. The PSMA-PET disease extent, categorized by PCGW3 clinical subtypes 2, is given in Table S1. The positivity rate stratified by PSA is given in Figure S3. There was a moderate, significant positive correlation between PSA at the time of PET and the presence of M1 disease (*p* = 0.05). Liver areas consistent with PCa metastases were found in 17/60 (28%) patients. Multiple (>1) liver lesions were seen in 11/17 (65%) of patients, and PSMA uptake in liver areas was increased in 14/17 (82%), reduced/absent in 1/17 (6%) and heterogeneous in 2 (12%) of patients (Table 2). Inter-reader agreement for liver metastases among readers was substantial, with a kappa of 0.762 (95% CI 0.757–0.767).

**Figure 3:**
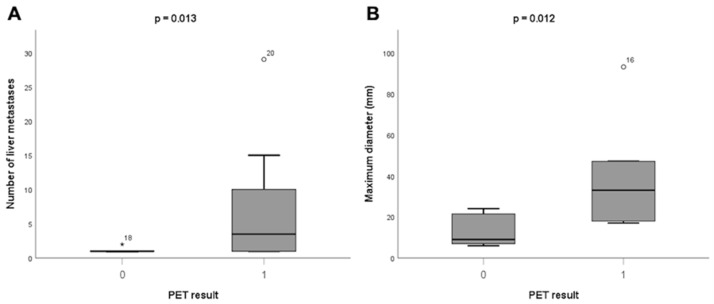
Boxplots for liver metastases characteristics at reference standard and PSMA-PET result: number of liver metastases (**A**) and maximum diameter (**B**). Notes: 0 = negative PET for liver metastases; 1 = positive PET for liver metastases; * = high extreme value, score is more than 3 IQR above quartile 3.

Performance of PSMA-PET for liver metastases. Compared to the reference standard, PSMA-PET was true positive in 14/17 (82%). PSMA-PET sensitivity was 0.58 (95% CI 0.37–0.78), specificity 0.92 (95% CI 0.76–0.98), positive predictive value (PPV) 0.82 (95% CI 0.57–0.96), negative predictive value (NPV) 0.77 (95% CI 0.61–0.88) and accuracy 0.78 (95% CI 0.66–0.88). There were 10/24 (42%) patients with PSMA-PET negative for liver metastases while positive according to a reference standard. Boxplots for liver metastases characteristics and PSMA-PET results are given in Figure 3. The median number of liver lesions confirmed at the reference standard was higher in patients with a PSMA-PET positive for hepatic disease compared to patients with a PSMA-PET negative within the liver (median 4 vs. 1 metastasis, *p* = 0.013; Figure 3). Moreover, the median maximum diameter of hepatic metastases on reference standard was higher in PSMA-PET positive liver compared to PSMA-PET negative liver (median 33 vs. 9 mm, *p* = 0.012; Figure 3). The mean SUVmax of all PET-positive liver metastases was 20 ± 6, compared to a mean SUVmax of the normal liver of 6 ± 3, resulting in a statistically significant difference (*p* < 0.001).

#### 3.2.3. False Positive PSMA-PET Findings

Three/sixty (5%) patients had a positive PSMA-PET for liver metastases ruled out by reference standard. One patient underwent a liver biopsy revealing focal hepatitis, whereas two patients performed ceCT showing a perfusion defect or the presence of hepatocarcinoma (Figure S4), respectively.

#### 3.2.4. Factors Associated with Liver Metastases

On multivariable regression analysis, PSA at the time of PSMA-PET was associated with the presence of liver metastases at reference standard (OR 1.03; 95% CI 1.01–1.06; *p* = 0.019). Similarly, PSMA-PET suggesting M1 disease within the liver was associated with the demonstration of liver metastases at reference standard (OR 21.09; 95% CI 4.67–128.56; *p* < 0.001). All other factors were not significantly associated with liver metastases (Table S2).

### 3.3. Radiomic Analysis

**Figure 4:**
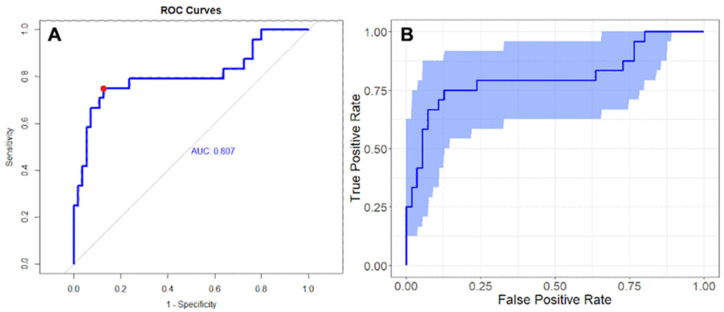
ROC curve of the best radiomic model in predicting the presence of liver metastases (AUC 0.807; specificity 0.87; sensitivity 0.75) (**A**) and result of Bootstrap simulation obtained with 1000 replications (95% CI 0.686–0.920) (**B**).

Radiomic analysis was performed on 59/60 (98%) patients because one patient had a PET/MRI and thus was excluded (Figure 1). Three CT-derived features and four PET-derived features were significantly associated with the presence of liver metastases at reference standard (Tables S3–S5 and Figure S5). At multivariable regression analysis, the model combining one CT feature, i.e., sphericity, and one PET features, i.e., inverse difference moment (Idm), had an AUC of 0.807 (95% CI 0.686–0.920) and reached a sensitivity and a sensibility of 0.75 and 0.87, respectively (Table 3 and Figure 4).

## 4. Discussion

### 4.1. [68Ga]Ga-PSMA-11 PET in CRPC Patients with Liver Metastases

Visceral involvement in PCa represents a negative prognostic factor with increased lethality for lung and liver metastases compared with bone and non-visceral involvement [19,20,21]. Imaging is thus essential for the identification of CRPC clinical subtypes [2]. The diagnostic performance of [68Ga]Ga-PSMA-11 PET for visceral metastases of CRPC patients has never been systematically assessed, even if PSMA-PET is becoming largely employed worldwide for multiple indications, e.g., diagnosis and primary staging of PCa [22,23]. The relatively moderate background uptake of [68Ga]Ga-PSMA-11 within the liver is a major limitation, as well as the presence of motion-induced blurring of the hepatic parenchyma during PET imaging acquisition for the evaluation of unfavorable hepatic metastases [9]. Several PSMA-ligands for PET imaging are available, and others under investigation, e.g., [18F]-DCFPyL and [18F]-PSMA-1007, even if the background uptake within the liver is constantly present, even if at several degrees of intensity.

### 4.2. Diagnostic Performance of PSMA-PET in the Detection of Liver Metastases Compared to Conventional Imaging/Liver Biopsy

Here we assess in a retrospective multicenter study the diagnostic performance of PSMA-PET for detecting liver metastases compared to ceCT, MRI, or liver biopsy. To date, this is the first study aiming at this, combining both a qualitative analysis and a radiomic approach. More than two-thirds of patients had advanced disease (bone and/or visceral disease) with previous CRPC systemic therapy. PSMA-PET demonstrated high specificity, positive predictive value, and reproducibility. With a specificity of 91.7% and a positive predictive value of 82.4%, areas of altered PSMA uptake demonstrated a good association with the presence of liver metastases. Notably, an inter-reader agreement was substantial among blinded and independent reads, despite the challenging above-mentioned characteristics of the liver parenchyma. This value is slightly more favorable compared to the inter-reader agreement reached among highly experienced readers when assessing visceral metastases in a multicenter study including 50 patients (Fleiss’ κ = 0.76 vs. 0.61, respectively) [24]. However, Fendler et al. did not differentiate liver metastases among the visceral metastases’ subgroup patients and reported data on a cohort of PC patients less extensively treated (80% during primary staging or biochemical recurrence/persistence). According to Damjanovic et al., the SUVmax of PSMA-positive liver metastases was significantly higher than that of the normal liver tissue [25], confirming that a certain number of liver metastases highly overexpress PSMA and are therefore directly detectable, as confirmed by the value of the inter-reader agreement. However, patients with PSMA-negative or mixed uptake in liver metastases were found. Most importantly, false negative results for liver lesions were found in our population, and this is a known limitation for PSMA-PET when assessing visceral metastases, leading to PET down-staging when compared to conventional imaging (e.g., ceCT or MRI) [8,11,25]. This result is against that one of Damjanovic and colleagues, where the majority of liver metastases highly overexpressed PSMA [25]. However, despite the similar number of patients with confirmed liver metastases between the two studies (26 vs. 18), the median PSA at the time of PET was significantly lower in the present study, i.e., 6 vs. 124 ng/mL, suggesting that differences in disease burden and stage of PC are not negligible between these two populations. An explanation for the different behavior of PSMA uptake within the liver could be the heterogeneity of phenotypes in metastases, predominantly the neuroendocrine dedifferentiation. In the majority of metastatic CRPC, neuroendocrine biomarker profiles, such as a progastrin-releasing peptide, neuron-specific enolase, and chromogranin-A, are abnormal [26]. Hepatic metastases are frequently associated with neuroendocrine characteristics, especially in CRPC patients with exposure to long-term androgen-ablating therapy [4,27], and a loss of PSMA expression is thus hypothesized [25,28]. Moreover, we found a statistically significant association between a false-negative PSMA-PET for liver metastases and the presence of only one lesion and the small diameter of the lesion, supporting the hypothesis that blurring respiratory motion-related and spatial resolution are intrinsic PET limitations. Consequently, it is very likely to miss small and single metastases in CRPC patients with PSMA-PET. Based on our false positive findings and the available literature, PSMA-avid differential diagnosis, such as vascular disorders (hemangiomas, perfusional defects) and hepatic diseases (hepatitis, hepatocarcinoma), should be considered when assessing the liver parenchyma with PSMA-PET [29,30,31,32]. Hybrid PET/CT and PET/MRI systems, with dedicated multi-phase acquisitions employing contrast, are supposed to increase the sensitivity for hepatic metastases in patients with advanced CRPC, however.

### 4.3. Potential Use of Radiamics

Radiomics has been developed for clinical problem solving, and radiomics features were extracted from an ROI, including the whole liver, to overcome the strong dependence of radiomic features from the applied segmentation methods [33]. Despite the relatively low number of patients, the multivariate analysis identified Idm, extracted from PSMA-PET, and sphericity, extracted from CT, as predictive for liver metastases. Regarding the identified features, the CT-based sphericity is a dimensionless measure independent of scale and orientation, which is a measure of the roundness of the shape of the target region relative to a sphere (ranging from 0 to 1, with 1 indicating a perfect sphere). It is correlated to compactness and spherical disproportion and is independent of liver size. So, this feature might reveal the difference in the liver structure in the presence of metastases. Idm is a measure of the local homogeneity of the image. A possible clinical explanation may be related to intra-tumoral heterogeneity in PSMA-expressing cells within liver lesions. This is an additional interesting finding since Idm is not largely affected by liver size. Moreover, Idm has already achieved less sensitivity in terms of coefficient of variation, percent deviation, level of image contrast, scanner model, and acquisition parameters [34]. Furthermore, texture features, such as Idm, provide a more descriptive measure of voxels arrangement and their relative neighborhood to each other, which is in line with the notion of disease heterogeneity. For these reasons, CT-based sphericity and PSMA-PET-based Idm are suggested as predictive features for liver metastases identification in the next future. However, the optimal thresholds of identified features should be further investigated and confirmed in larger datasets and/or heterogeneous phantoms [35].

### 4.4. Strengths of the Study

There are several strengths of this study. Our study is strengthened by its multicenter design and implementation of blinded reads and independent liver lesion validation. Image interpretation was defined by a statistical consensus of trained, independent, and blinded readers. Validation was performed by reference imaging standard criteria or liver biopsy. Lastly, PET read was associated with a radiomic analysis of the liver.

### 4.5. Limitations

The present study has, however, several limitations. Firstly, it is a retrospective design. Secondly, the six-month maximum timeframe between PSMA-PET and the reference standard is relatively wide for CRPC patients, even if without any change of treatment, potentially affecting results interpretation. Then, the cohort of patients is relatively small, with 60 patients and only 24 with liver metastases. Also, only a few patients were validated through liver biopsy, potentially leading to an underestimation of the exact number of liver metastases. Lastly, the lack of clinical follow-up with the consequent absence of oncological outcomes is noteworthy.

## 5. Conclusions

[68Ga]Ga-PSMA-11 PET demonstrated high specificity, positive predictive value, and reproducibility compared to conventional imaging and liver biopsy when assessing unfavorable liver metastases in CRPC patients. Single and dimensionally smaller metastases are at higher risk of being missed. However, nuclear medicine physicians should carefully assess the liver parenchyma on PET images, especially in those patients at higher risk for liver metastases. Moreover, radiomic features may aid the physician in recognizing them. Further studies are warranted to test these findings in a prospective and larger cohort of patients and to correlate PSMA expression within the liver with histopathology.

## Figures and Tables

**Table 1:** Patient characteristics.

	Mean ± SD	Median	IQR
Age (years)	70 (9)	72	64–75
PSA at time of PET scan (ng/mL)	80.5 (179.9)	6.3	1.6–45.3
∆ date PET—date reference standard (months)	3 (3)	1	0–5
	Frequency		**%**
≥pT3a	31/39		80
pN1	20/41		49
ISUP Grade Group ≥ 4	30/51		57
Previous therapies			
Radical prostatectomy	44/60		73
External beam radiation therapy	4/60		7
Hormonal therapy	60/60		100
Docetaxel	12/60		20
Cabazitaxel	6/60		10
Abiraterone/Enzalutamide/Apalutamide	23/60		38
Radium-223	3/60		5
Palliative RT	2/60 12/60		3 20
PSMA-RLT On-going hormonal therapy at the time of PET	43/60		72

Notes: SD = standard deviation; IQR = interquartile range; ISUP = International Society of Urological Pathology; PSMA-RLT = PSMA-radioligand therapy with 177Lu.

**Table 2:** PSMA-PET liver findings at qualitative analysis including both true positive and false positive lesions (*n* = 17).

	Single Lesion (%)	Multiple Lesions (%)	Total (%)	Median SUVmax Lesion (IQR)	Median SUVmax Liver Parenchyma (IQR)
Absent uptake	1 (6)	-	1 (6)	4 (4–4)	11 (11–11)
Increased uptake	5 (29)	9 (53)	14 (82)	20 (18–24)	6 (4–8)
Heterogeneous uptake	-	2 (12)	2 (12)	25 (12–22)	6 (6–7)
Total	6 (35)	11 (65)	17 (100)	20 (17–24)	6 (4–8)

**Table 3:** GLM regression model for radiomic feature in predicting the presence of liver metastases.

Variable	Estimate	Standard Error	T Value	Pr(>|t|)	Odds Ratio	Sign 95%
(Intercept)	2.53	0.63	3.99	0.00015	12.54	[4.42; 35.56]
CT_Sphericity	−3.99	1.05	−3.80	0.00029	0.018	[0.003; 0.10]
PET_Idm	1.67	0.57	2.93	0.00449	5.29	[2.08; 13.48]

## Data Availability

Data supporting the reported results can be obtained from the corresponding author.

## Supplementary Materials

The following supporting information can be downloaded at: https://www.mdpi.com/article/10.3390/cancers14225680/s1, Figure S1. Patient-based analysis of the localization of liver metastases, according to liver segments and reference standard; Figure S2. Boxplots displaying distribution of Gleason score sum, PSA at time of PSMA-PET and number of previous lines of therapy for CRPC and presence (1) or absence (0) of liver metastases at reference standard; Figure S3. PSMA-PET positivity rate on patient basis stratified by PSA and number of lesions; Figure S4. Case example of false positive PSMA-PET for liver metastases; Table S1. PSMA-PET for CRPC staging categorized by PCWG3 clinical subtype and PROMISE stage; Figure S5. Correlation analysis for significant CT (a) and PSMA-PET radiomic features (b); Table S2. Logistic regression model for predicting presence of liver metastases; Table S3. CT (n = 6) and PSMA-PET (n = 35) radiomic features predicting presence of liver metastases at univariate analysis model; Table S4. Low correlated radiomic features included in the multivariate analyses; Table S5. High correlated radiomic features.
