# Supplementary material for: PSMA PET for the Evaluation of Liver Metastases in Castration-Resistant Prostate Cancer Patients: A Multicenter Retrospective Study
Source: Cancers (Basel). 2022 Nov 18;14(22):5680. doi: 10.3390/cancers14225680 (PMC9688898; doi:10.3390/cancers14225680)

SUPPLEMENTAL MATERIAL

Table S1. PSMA-PET for CRPC staging categorized by PCWG3 clinical subtype and PROMISE stage.

	**PSMA-PET (%)**<br>**n = 60**	**Liver metastases confirmed at reference standard (%)**<br>**n = 24**
**Non-metastatic**	5 (8)	1 (4)
**Local (T/Tr only)**	4 (7)	2 (8)
**Nodal (N1/M1a ± T/Tr)**	10 (17)	1 (4)
N1 only	3 (5)	-
M1a only	3 (5)	1 (4)
**Bone (any M1b)**	19 (32)	5 (21)
**Visceral (any M1c)**	22 (37)	15 (63)
Liver	17 (28)	14 (58)

Table S2. Logistic regression model for predicting presence of liver metastases.

	**β**	**SE β**	**OR**	**95% CI**	**p value**
**(Costant)**	-2.024	0.580	na	na	$p < 0.001$
**PSA PET (ng/mL)***	0.029	0.012	1.03	1.01 – 1.06	$p = 0.019$
**PSMA-PET result**	3.048	0.829	21.09	4.67 – 128.56	$p < 0.001$

Notes: na=not available.

* PSA outliers were excluded (n=2).

Table S3. CT (n=6) and PSMA-PET (n=35) radiomic features predicting presence of liver metastases at univariate analysis model (only p<0.05 are shown).

**CT**		**PSMA-PET**											
	**p value**		**p value**		**p value**		**p value**		**p value**		**p value**		**p value**
Sphericity	0.000823	Surface Volume Ratio	0.003094	Total Energy	0.026237	Gray Level Variance .1	0.030874	Difference Average	0.036195	Inverse Variance	0.042278	Large Area Emphasis	0.045631
Surface Volume Ratio	0.005208	Sphericity	0.004498	Gray Level Variance .2	0.028475	Busyness	0.031711	Mean Absolute Deviation	0.04016	Long Run High Gray Level Emphasis	0.043372	Idm	0.046797
Elongation	0.04428	Cluster Prominence	0.016222	Contrast	0.030056	Difference Entropy	0.035257	Strength	0.04016	High Gray Level Zone Emphasis	0.043372	Id	0.047988
Kurtosis	0.045409	Complexity	0.018744	Maximum	0.030056	Cluster Tendency	0.035257	High Gray Level Emphasis	0.041208	Autocorrelation	0.044489	Entropy	0.047988
Maximum 2D Diameter Slice	0.046616	Sum Squares	0.023494	Gray Level Variance	0.030874	Range	0.035257	Short Run High Gray Level Emphasis	0.041208	Small Area High Gray Level Emphasis	0.044489	Zone Variance	0.049205
10 Percentile	0.04757	Difference Variance	0.025528	Variance	0.030874	Small Dependence High Gray Level Emphasis	0.036195	High Gray Level Run Emphasis	0.041208	Sum Entropy	0.045631		

Table S4. Low correlated radiomic features included in the multivariate analyses.

**CT low correlated features**	
2	Sphericity
4	10Percentile
**PET low correlated features**	
1	SurfaceVolumeRatio
2	Autocorrelation
3	ZoneVariance

Table S5. High correlated radiomic features.

**CT high correlated features**	
1	SurfaceVolumeRatio
2	Maximum2DDiameterSlice
3	Elongation
4	Kurtosis
**PET high correlated features**	
1	GrayLevelVariance.2
2	GrayLevelVariance.1
3	GrayLevelVariance
4	Variance
5	SumSquares
6	Entropy
7	SmallDependenceHighGrayLevelEmphasis
8	MeanAbsoluteDeviation
9	SmallAreaHighGrayLevelEmphasis
10	ClusterTendency
11	SumEntropy
12	HighGrayLevelZoneEmphasis
13	ShortRunHighGrayLevelEmphasis
14	HighGrayLevelEmphasis
15	HighGrayLevelRunEmphasis
16	LongRunHighGrayLevelEmphasis
17	TotalEnergy
18	ClusterProminence
19	Busyness
20	Contrast
21	DifferenceVariance
22	DifferenceAverage
23	DifferenceEntropy
24	Maximum
25	Complexity
26	Range
27	Id
28	Strength
29	Idm
30	InverseVariance
31	LargeAreaEmphasis
32	Sphericity

Figure S1. Patient-based analysis of the localization of liver metastases, according to liver segments and reference standard. Percentages indicate the proportion of study patients in whom liver metastases were localized within the respective liver segment.

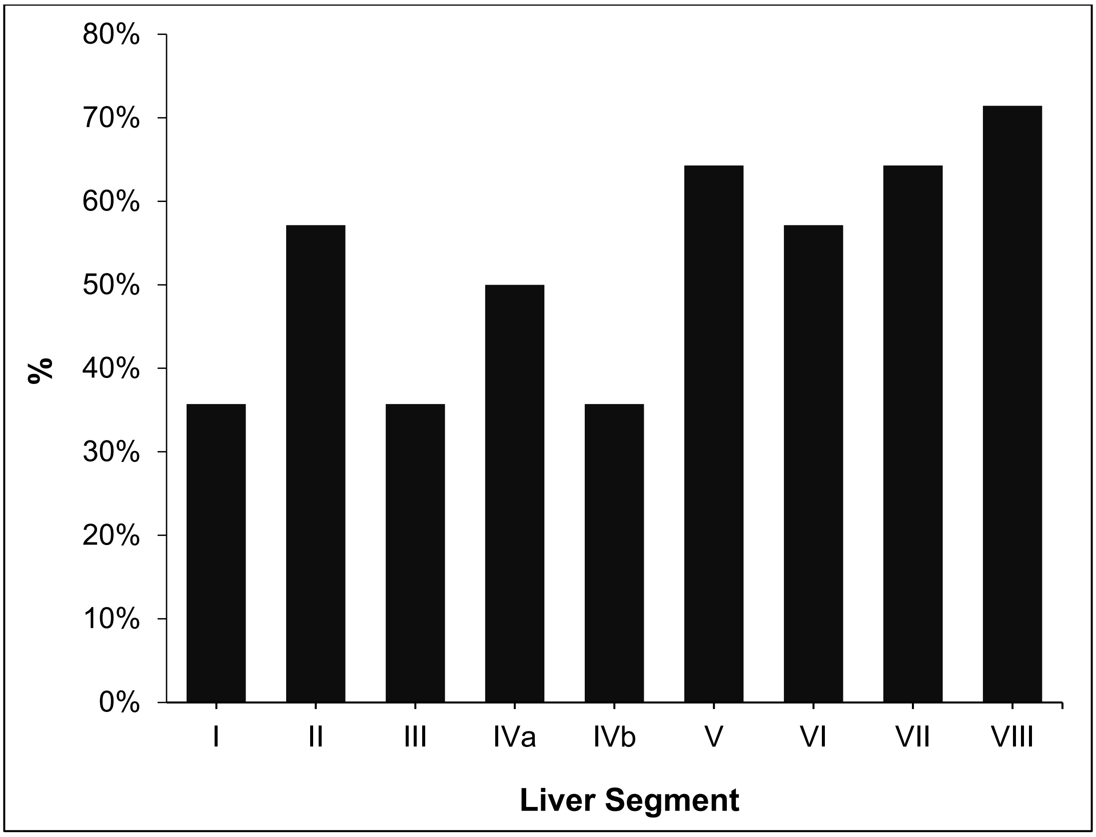

Figure S2. Boxplots displaying distribution of Gleason score sum, PSA at time of PSMA-PET and number of previous lines of therapy for CRPC and presence (1) or absence (0) of liver metastases at reference standard.

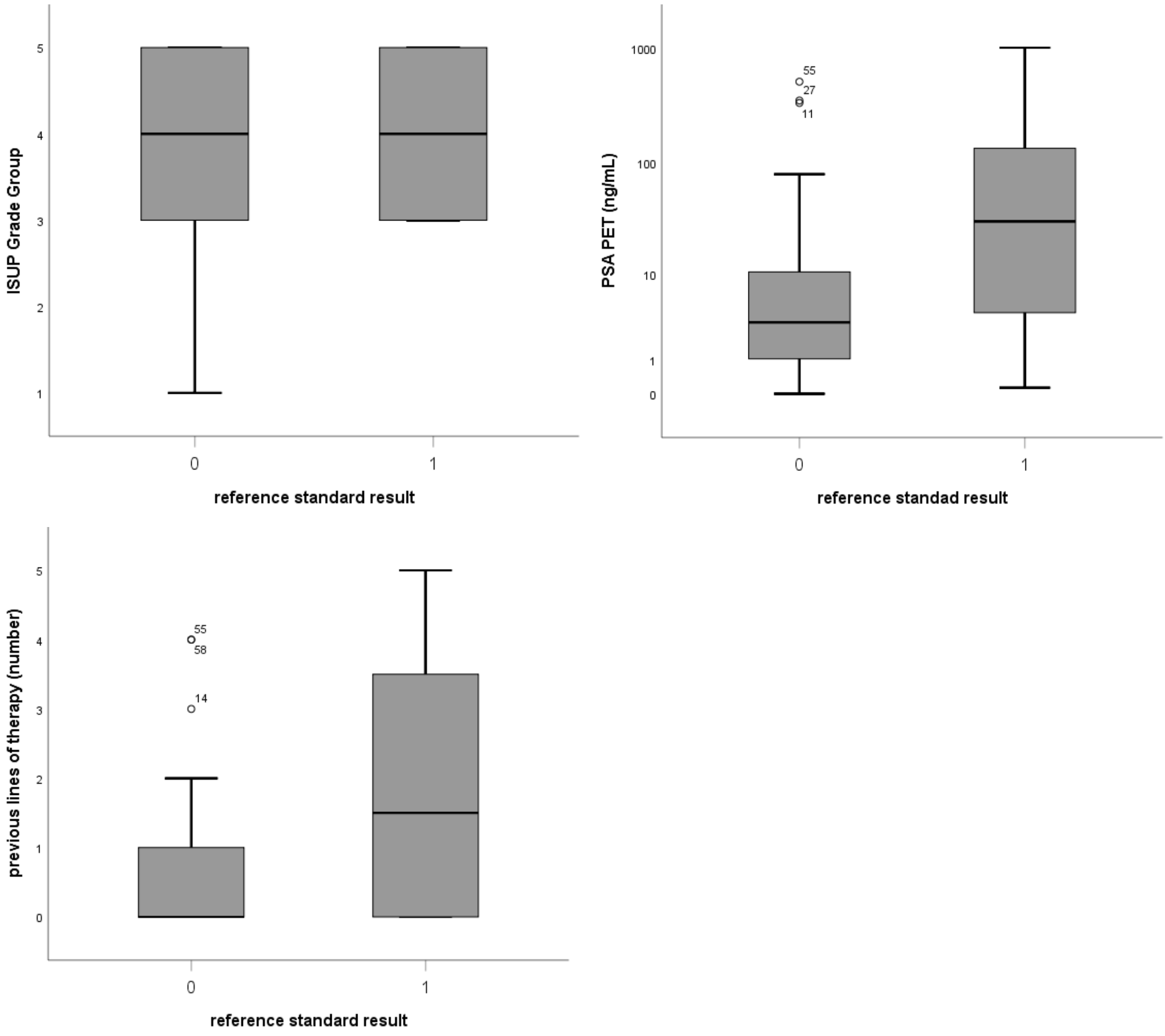

Notes: 0 = negative reference standard for liver metastases; 1 = positive reference standard for liver metastases.

Figure S3. PSMA-PET positivity rate on patient basis stratified by PSA and number of lesions.

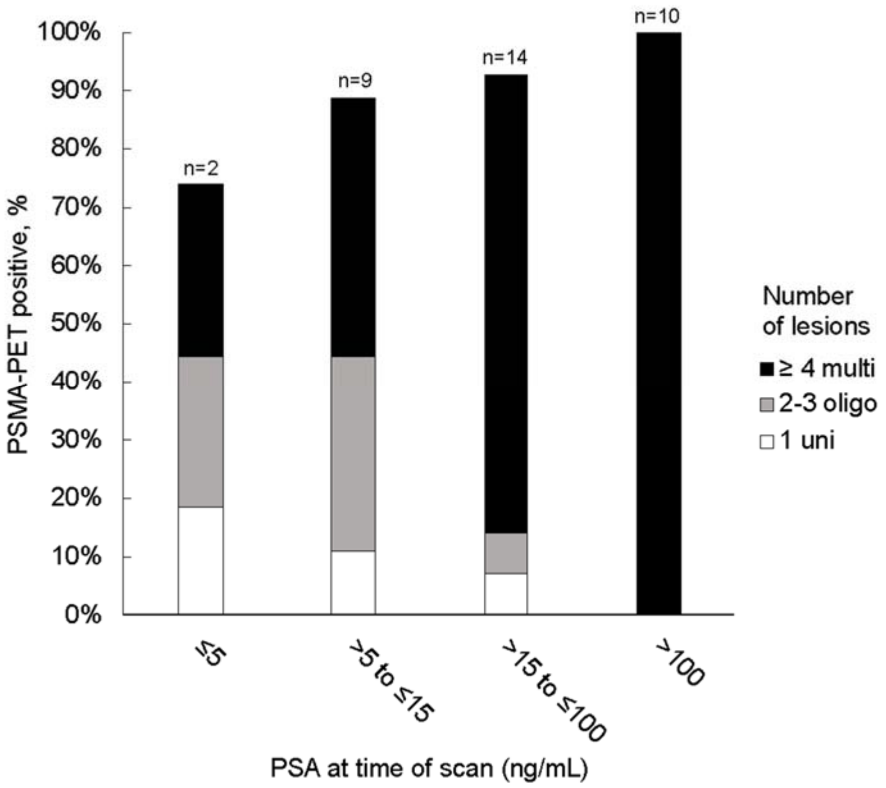

Figure S4. Case example of false positive PSMA-PET for liver metastases.

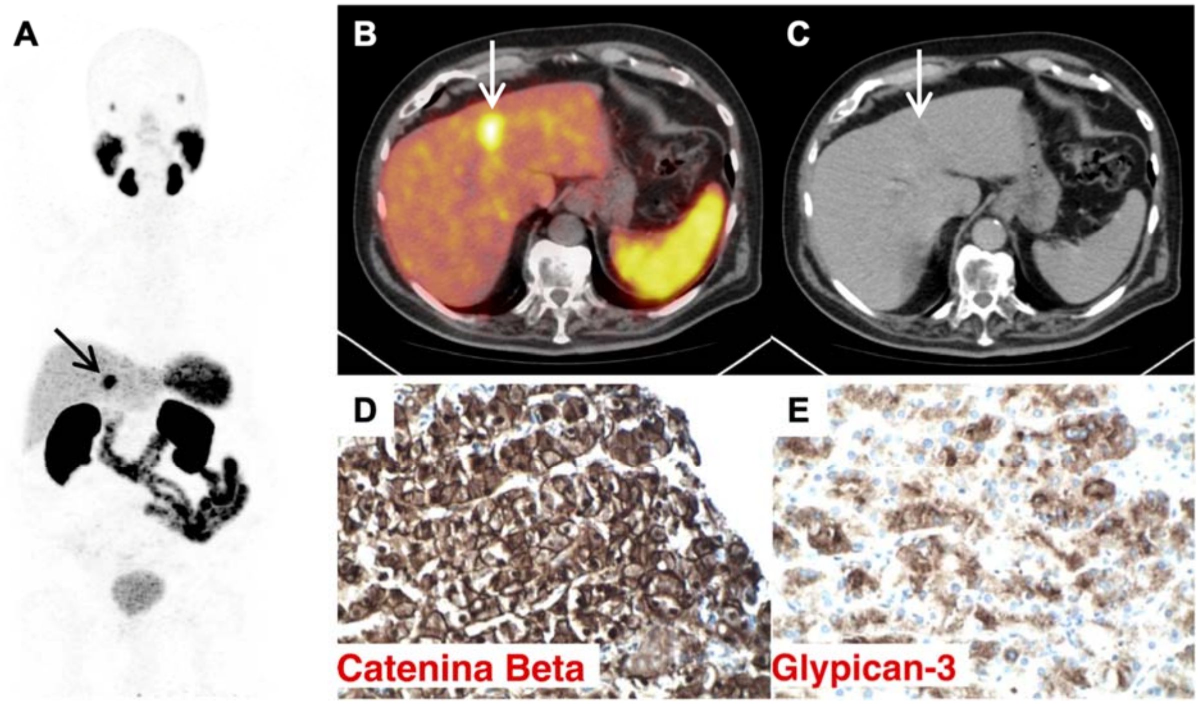

82-year-old man diagnosed with high-risk PC treated with radical prostatectomy and pelvic lymph node dissection in 2018, pT3a pN0, R1, ISUP 3. Hormonal therapy was started with progression under treatment and diagnosis of CRPC. Due to an increasing PSA, he underwent a PSMA-PET in July 2020 with PSA of 0.14 ng/mL. Focal PSMA uptake was demonstrated in the IV hepatic segment (A and B), whereas the low-dose CT-part of the scan showed a hypodense area (C). With known PET-information, the patient underwent a multi-phase CT. Histopathology performed five months later showed the presence of a well-differentiated hepatocarcinoma with ki-67 of 10%, positive for Catenina Beta (D), Glypican-3 (E), Arginase-1 and Glutamine synthetase.

Figure S5. Correlation analysis for significant CT (**a**) and PSMA-PET radiomic features (**b**). Cut-off was set at 0.6.

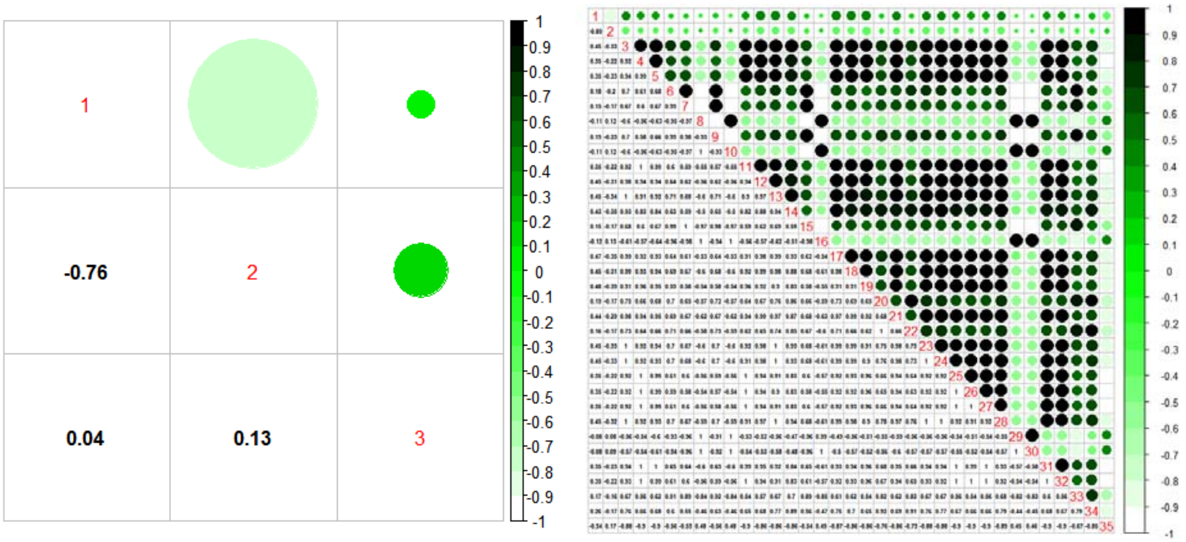

Supplement: Supplementary file 1 [file cancers-14-05680-s001.zip › cancers-1948594-supplementary.pdf]
